# Cardiac magnetic resonance imaging-indeterminate/negative cardiac sarcoidosis revealed by ^18^F-fluorodeoxyglucose-positron emission tomography: two case reports and a review of the literature

**DOI:** 10.1186/s13256-017-1453-6

**Published:** 2017-10-20

**Authors:** S. C. Sasson, R. Russo, T. Chung, G. Chu, I. Hunyor, J. Williamson, A. Murad, A. Kane, S. Riminton, S. Limaye

**Affiliations:** 1 Department of Clinical Immunology, Concord Hospital, Level 6, Hospital Rd, Concord, Sydney, NSW 2139 Australia; 2 Sydney Medical School, University of Sydney, Sydney, Australia; 3 Department of Nuclear Medicine, Concord Hospital, Sydney, Australia; 4 Department of Cardiology, Concord Hospital, Sydney, Australia; 5 Department of Immunology, Liverpool Hospital, Sydney, Australia; 6 Department of Cardiology, Royal Prince Alfred Hospital, Sydney, Australia; 7 Department of Respiratory Medicine, Liverpool Hospital, Sydney, Australia; 8 South Western Sydney Clinical School, University of New South Wales, Sydney, Australia

**Keywords:** Cardiac sarcoidosis, CMR, FDG-PET

## Abstract

**Background:**

Sarcoidosis is an inflammatory disorder of immune dysregulation characterized by non-caseating granulomas that can affect any organ. Cardiac sarcoidosis is an under-recognized entity that has a heterogeneous presentation and may occur independently or with any severity of systemic disease. Diagnosing cardiac sarcoidosis remains problematic with endomyocardial biopsies associated with a high risk of complications. Several diagnostic algorithms are currently available that rely on histopathology or clinical and radiological measures. The dominant mode of diagnostic imaging to date for cardiac sarcoidosis has been cardiac magnetic resonance imaging with gadolinium enhancement.

**Case presentations:**

We report the cases of two adult patients: case 1, a 50-year-old white man who presented with severe congestive cardiac failure; and case 2, a 37-year-old white woman who presented with complete heart block. Both patients had a background of untreated pulmonary sarcoidosis. Cardiac magnetic resonance imaging did not show evidence of sarcoidosis in either patient and both proceeded to ^18^F-fluorodeoxyglucose-positron emission tomography scans that were highly suggestive of cardiac sarcoidosis. Both patients were systemically immunosuppressed with orally administered prednisone and methotrexate and had subsequent improvement by clinical and nuclear medicine imaging measures.

**Conclusions:**

Current consensus guidelines recommend all patients with sarcoidosis undergo screening for occult cardiac disease, with thorough history and examination, electrocardiogram, and transthoracic echocardiogram. If any abnormalities are detected, advanced cardiac imaging should follow. While cardiac magnetic resonance imaging identifies the majority of cardiac sarcoidosis, early disease may not be detected. These cases demonstrate ^18^F-fluorodeoxyglucose-positron emission tomography is warranted following an indeterminate or normal cardiac magnetic resonance imaging if clinical suspicion remains high. Unidentified and untreated cardiac sarcoidosis risks significant morbidity and mortality, but early detection can facilitate disease-modifying immunosuppression and cardiac-specific interventions.

## Background

Sarcoidosis is a multisystem granulomatous disease of unknown etiology, with early descriptions dating back to 1869 [1]. Current pathophysiology models propose an aberrant immune reaction to an unknown antigen in genetically susceptible hosts [2, 3]. Sarcoidosis has a lifetime prevalence of 4 to 40/100,000 in whites and up to three times that in other racial groups such as African-Americans [4]. Sarcoidosis can affect any organ system but most commonly affects the lymph nodes and lungs and is non-life-threatening. Non-caseating, non-necrotic granulomas are the histological hallmark; however, chronic disease may lead to fibrosis.

Unlike systemic or pulmonary sarcoidosis, cardiac sarcoidosis (CS) may present acutely and is associated with a poorer prognosis and higher mortality, largely related to the increased risk of sudden cardiac death [5]. In addition, it is a highly under-recognized entity. While studies suggest CS is diagnosed in 2 to 7% of patients with sarcoidosis [6], autopsy studies demonstrate cardiac involvement in up to 25% [7, 8], with more than half clinically occult at time of death [4, 9]. Imaging studies aimed at detecting asymptomatic CS in patients with sarcoidosis suggest involvement in up to 55% (summarized in [10]). An accurate estimate of CS prevalence is unclear due to a lack of standardized diagnostic criteria. Ethnic variation exists, with CS evident in 68% of Japanese but 14% of white patients with sarcoidosis [11, 12]. Sarcoid granulomas are most frequently detected in the myocardium but can also occur in the endocardium and pericardium. Commonly effected areas include the basal ventricular septum, left ventricular (LV) free wall, papillary muscles, and right ventricle [6]. Importantly, CS accounts for a significant proportion (10 to 25%) of sarcoid deaths in the US population [9] and even higher (50%) in Japanese populations [7, 11] highlighting the need for early detection and intervention [6].

Clinical presentation of CS is heterogeneous ranging from asymptomatic, pre-syncope, syncope, atrial arrhythmias, ventricular dysfunction including congestive cardiac failure (CCF), and sudden cardiac death. In a study of 42 patients with CS the most common presenting symptoms were atrial-ventricular (AV) block (50%), LV heart failure (40%), syncope (31%), and palpitations (17%) [13]. The mechanisms underlying conduction abnormalities and ventricular dysfunction are directly related to granulomatous inflammation [9, 14]. Up to 65% of CS occurs without any evidence of extra-CS [15].

Current American Thoracic Society and European Respiratory Society guidelines recommend yearly screening for asymptomatic CS in any patient with sarcoidosis. At the minimum, this should include a directed cardiovascular history and examination, 12-lead electrocardiogram (ECG) and transthoracic echocardiogram (TTE) [10]. A Holter monitor may also be of use. Abnormalities in any one of these screening variables have a sensitivity of 100% and specificity of 87% for the diagnosis of CS [12]. ECG abnormalities are detected in 50% of patients with sarcoidosis and may include: pathological Q waves in two or more leads; fragmentation of QRS complex; complete, left, or right bundle branch blocks; sustained second or third degree AV node block; and sustained or non-sustained ventricular tachycardia (VT). The value of a TTE is largely in its negative predictive value as it has a sensitivity of 25% and specificity of 95% for detection of CS [10]. While TTE cannot detect infiltrative disease, it can identify impaired LV ejection fraction (LVEF), regional wall motion abnormalities, LV wall thickness or basal thinning, and/or aneurysm formation. The Heart Rhythm Society (HRS) expert consensus statement recommends proceeding to cardiac imaging with either cardiac magnetic resonance imaging (CMR) or ^18^F-fluorodeoxyglucose-positron emission tomography (FDG-PET) if any of the above are abnormal [10].

There is no validated biomarker for the screening of CS and while angiotensin-converting enzyme (ACE), lysozyme, high-sensitivity troponin, brain natriuretic peptide (BNP), and urinary calcium may all be elevated in patients with CS the diagnostic sensitivity for each remains low [15–17].

CMR combines excellent spatial resolution with myocardial characterization and has been the main imaging modality to date for identifying CS. The detection of delayed enhancement not consistent with coronary artery distribution determines the presence of scar tissue that is associated with CS [18]. CMR can also detect myocardial edema on T2-weighted images and has the additional advantage of detecting other infiltrative cardiac diseases if present. CMR has a relatively high sensitivity (76 to 100%) and specificity (78 to 92%) for the detection of CS [19–21], but cannot be performed in patients with some implantable cardiac devices. The presence and extent of delayed enhancement on CMR is inversely correlated to markers of LV function [22, 23] and is associated with a hazard ratio (HR) of 33.9 for cardiac events and 31.6 for lethal events [24–26]. Importantly, the extent of delayed enhancement on CMR is negatively correlated to response to immunosuppression, presumably because fibrotic tissue is less amenable to treatment [26]. Therefore, a modality such as FDG-PET that detects early disease and response to treatment would have significant advantages.

There are no widely accepted standard diagnostic criteria for CS due to lack of randomized controlled trial evidence of prospective value [21]. Endomyocardial biopsies are not recommended as standard of care for diagnosis as they have low sensitivity (25%) due to the patchy nature of disease and risk significant complications [27, 28]; however, image-guided biopsy may improve their yield.

Two commonly used algorithms are the Japanese Ministry of Health and Welfare (JMHW) 2007 criteria (see Table 1 [29]) and the HRS 2014 expert consensus statement (see Table 2 [10]) which are closely associated with the World Association for Sarcoidosis and Other Granulomatous Disorders (WASOG) criteria updated in 2014 [30]. The JMHW criteria and HRS consensus statement are similar in that both outline two pathways to the diagnosis of CS. The first is a histological diagnosis from myocardial tissue showing non-caseating granulomas and negative organism stain. The second pathway involves clinical diagnosis of probable CS and must satisfy several criteria (see Tables 1 and 2). The HRS consensus statement is the more recent algorithm and is argued as having increased sensitivity due to the inclusion of additional criteria of: steroid or immunosuppressive responsive cardiomyopathy or heart block, and patchy uptake on cardiac FDG-PET. The two algorithms have not been compared in clinical studies [21].

**Table 1:** Abbreviated Japanese Ministry of Health and Welfare guidelines for the diagnosis of cardiac sarcoidosis

Diagnosis group	Major criteria	Minor criteria
*Histological* Non-caseating granuloma on myocardial biopsy	N/A	N/A
*Clinical* Myocardial biopsy does not confirm cardiac sarcoidosis. *and* Two major criteria *or* One major and two minor criteria present.	• Advanced atrioventricular block • Basal thinning of the intraventricular septum • Positive cardiac ^67^Ga uptake • LVEF < 50%	• Abnormal ECG findings • Abnormal TTE findings • Nuclear medicine perfusion defect by ^201^Tl or ^99^Tc myocardial scintigraphy • Delayed enhancement of myocardium on MRI-GAD • Endomyocardial biopsy showing interstitial fibrosis or monocyte infiltration

For complete list of electrocardiogram and transthoracic echocardiogram findings please see full reference [29]. *ECG* electrocardiogram, *Ga* gallium, *GAD* gadolinium, *LVEF* left ventricular ejection fraction, *MRI* magnetic resonance imaging, *N/A* not applicable, *Tc* technetium, *Tl* thallium, *TTE* transthoracic echocardiogram

**Table 2:** Heart Rhythm Society criteria for the diagnosis of cardiac sarcoidosis (adapted from Birnie *et al*. [10])

Diagnosis group
*Histological* Non-caseating granuloma on myocardial biopsy without alternative cause
*(Probable) Clinical* Histological diagnosis of extra-cardiac sarcoidosis *and* One of the following: • Steroid responsive cardiomyopathy or heart block • Unexplained LVEF < 40% • Unexplained sustained VT • Advanced heart block • Patchy uptake on cardiac PET • Late GAD enhancement on cardiac MRI • Positive gallium uptake *and* Other causes for the cardiac manifestations have been excluded.

*GAD* gadolinium, *LVEF* left ventricular ejection fraction, *MRI* magnetic resonance imaging, *PET* positron emission tomography, *VT* ventricular tachycardia

Here we report two cases of adult patients: one who presented with severe CCF and one who presented with complete heart block (CHB). Both patients had a background of untreated pulmonary sarcoidosis. CMR did not show evidence of sarcoidosis in either patient and both went on to have FDG-PET scans that facilitated the diagnosis of CS. These cases illustrate the difficulty in diagnosing CS due to heterogeneous presentations and a lack of unified diagnostic criteria. They also highlight the utility of FDG-PET for diagnosis of CS in patients where CMR is indeterminate or negative, but where clinical suspicion for disease remains high.

## Case presentations

### Case 1

A 50-year-old white man presented with a 3-month history of shortness of breath and pleuritic chest pain not responding to antibiotics for community-acquired pneumonia. His history included pulmonary sarcoidosis diagnosed 2 years prior following a chest X-ray (CXR) that incidentally demonstrated perihilar lymphadenopathy. The diagnosis was histologically confirmed by endobronchial ultrasound (EBUS) and biopsy-proven granulomatous inflammation. The sarcoidosis was not initially treated. He reported 2 years of disabling fatigue and had also had an episode of syncope 1 year prior when he stood suddenly from a prolonged supine position. He was assessed in an emergency department where a normal neurological examination was documented. ECG showed sinus rhythm with ventricular ectopic beats. He was discharged home with a provisional diagnosis of orthostatic hypotension. At current presentation he was afebrile and hemodynamically stable with arterial oxygen saturation (SaO_2_) of 95% on room air and with a respiratory rate of 26 breaths/minute. Auscultation of his chest revealed decreased air entry bilaterally with inspiratory crepitations. A CXR showed cardiomegaly with bilateral pleural effusions and reticulonodular opacification consistent with pulmonary edema. An ECG showed sinus rhythm with frequent ventricular ectopic beats and ventricular bigeminy, with no detected AV node block. A bedside TTE revealed a poor LVEF of 10 to 15% (normal range 55 to 70%) and small pericardial effusion with no basal thinning of the intraventricular septum. Blood tests were largely unremarkable with full blood count, renal and liver function tests, fasting blood sugar level (BSL), and electrolytes all within normal limits. A high sensitivity troponin was mildly elevated at 18 ng/L (reference range, RR < 14 ng/L) and C-reactive protein (CRP) was mildly elevated at 5.1 mg/L (RR < 5 mg/L); subsequently an N-terminal pro-hormone of BNP was also elevated at 593 pmol/L (RR < 13 pmol/L).

**Fig. 1:**
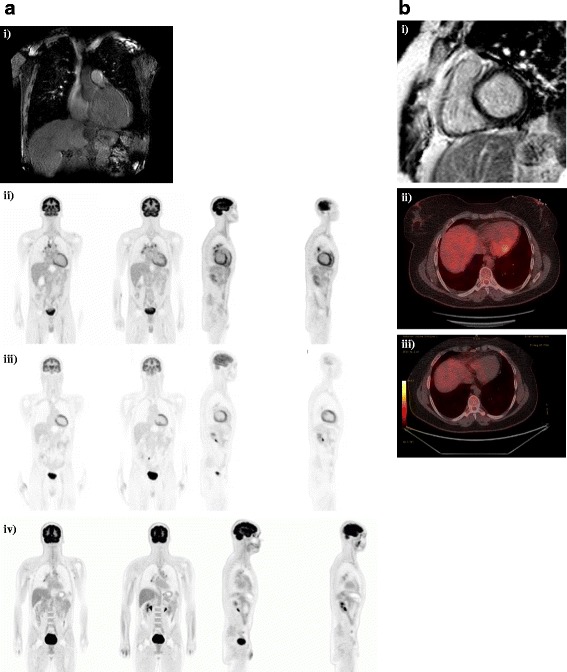
Cardiac magnetic resonance imaging with gadolinium and ^18^F-fluorodeoxyglucose-positron emission tomography at time of diagnosis and during follow-up. Advanced imaging for **a** Patient 1 shows **i** cardiac magnetic resonance imaging reported in the clinical setting as within normal limits. Re-review for the purposes of publication found the scan was of insufficient quality to accurately detect presence of delayed enhancement. **ii** ^18^F-fluorodeoxyglucose-positron emission tomography at time of presentation demonstrates abnormal heterogeneous and moderate-to-markedly increased metabolism in hilar and mediastinal nodes consistent with cardiac sarcoidosis, as well as cardiomegaly and diffuse uptake in both ventricles and the right atrium. **iii** Progress ^18^F-fluorodeoxyglucose-positron emission tomography following 3 months’ treatment with prednisone and methotrexate demonstrates response to treatment with a reduction in the size and metabolism of the hilar and mediastinal lymph nodes. In addition, the heart is smaller and the increased uptake seen in the right ventricle and the right atrium on the initial scan has resolved, although there is persistent metabolism in the septum. **iv** Progress ^18^F-fluorodeoxyglucose-positron emission tomography following 17 months of immunosuppression demonstrates response to treatment with complete resolution of abnormal metabolism in the myocardium, but persisting areas of avidity in the mediastinal and hilar lymph nodes. **b** Patient 2 shows **i** cardiac magnetic resonance imaging with no abnormalities detected at presentation. **ii** ^18^F-fluorodeoxyglucose-positron emission tomography at time of presentation demonstrates abnormal active sites of focal myocardial inflammation in the basal anteroseptum, basal septum, and inferior walls that was consistent with cardiac sarcoidosis. **iii** Progress ^18^F-fluorodeoxyglucose-positron emission tomography following 6 months of immunosuppression with prednisone and methotrexate demonstrates resolution of previously abnormal focal increased ^18^F-fluorodeoxyglucose accumulation in the left ventricle consistent with resolution of areas of inflammation

He was admitted to our coronary care unit with severe dilated cardiac myopathy and LV failure with a provisional diagnosis of post-viral myocarditis. He was treated with low-dose frusemide and an ACE inhibitor. Serology for human immunodeficiency virus (HIV), hepatitis B virus (HBV), hepatitis C virus (HCV), and varicella zoster virus (VZV) were all non-reactive. Interferon-gamma release assay (IGRA) for *Mycobacterium tuberculosis* (MTB) was negative. CMR demonstrated severe dilated non-ischemic cardiomyopathy with a LVEF of 17%. No focal areas of abnormal gadolinium (GAD) enhancement were found in the myocardium, which was reported as within normal limits (Fig. 1ai). It should be noted that subsequent review by a third party with expertise in CMR for the purpose of publication found the scan to be indeterminate based on technical reasons, and this is discussed further below. Dermatology and Ophthalmology review and examination found no evidence of cutaneous or ocular sarcoidosis. Additional markers for sarcoidosis were unrewarding including: ACE, 16 U/L (RR 8 to 64 U/L); 1,25 dihydroxyvitamin D, 54 pmol/L (RR 60 to 100); and urine calcium, 1.9 mmol/L. Autoimmune serology including antinuclear antibodies (ANA), extractable nuclear antigens (anti-ENA), antineutrophil cytoplasmic antibodies (ANCA), cyclic citrullinated peptide (anti-CCP), double-stranded DNA (dsDNA), C3, C4, and rheumatoid factor were all within normal limits.

On Day 7 our patient developed right upper quadrantanopia and investigations revealed left posterior cerebral artery (PCA) territory infarct secondary to thrombus. He underwent acute thrombolysis for probable cardiac thromboembolism and symptoms gradually resolved. On Days 7 and 9 he demonstrated short runs of non-sustained VT on cardiac monitoring.

He proceeded to FDG-PET following a 6-hour fast, to investigate for underlying CS. This showed heterogeneous and moderate-to-marked increased metabolism in hilar and mediastinal nodes consistent with sarcoidosis, as well as cardiomegaly and diffuse uptake in both ventricles and his right atrium. Although not classic for CS, where uptake is usually patchy, CS could not be excluded (Fig. 1aii). Endomyocardial biopsy was discussed and considered too great a risk in the setting of low LVEF and recent thrombolysis. He was commenced on systemic immunosuppression with prednisone 1 mg/kg orally administered daily and methotrexate. He was discharged on the above immunosuppression together with warfarin, bisoprolol, frusemide, ivabradine, magnesium aspartate, ramipril, and spironolactone. The decision for automated implantable cardiac defibrillator (AICD) was deferred and out-patient cardiac rehabilitation and transplant referral arranged.

A review at 2 weeks demonstrated a remarkable recovery with increased LVEF to 29% (from 10 to 15%). Our patient self-reported energy levels higher than at any time in the preceding 2 years, prompting a return to full-time work. Three months after the initial presentation he presented with worsening CCF and was found to be in atrial fibrillation (AF). He underwent successful direct current (DC) cardioversion and was commenced on sotalol. A progress FDG-PET at that time demonstrated response to treatment with a reduction in the size and metabolism of the hilar and mediastinal lymph nodes. In addition, his heart was smaller and the increased uptake seen in the right ventricle and the right atrium on the initial scan had resolved, although there was still persistent metabolism in the septum (Fig. 1aiii). The LVEF progressively improved from 47% at 4 months to 53% at 10 months. A progress FDG-PET at 17 months found no regions of increased metabolism in the myocardium, but persisting areas of avidity in his mediastinal and hilar lymph nodes (Fig. 1aiv). He remains clinically well and asymptomatic from systemic sarcoidosis or CS. Cardiac transplantation is no longer a consideration.

### Case 2

A 37-year-old white woman presented to an out-patient Cardiology unit for investigation of dizziness. Following a review a 24-hour Holter monitor found her to be in CHB. This occurred on a background of pulmonary sarcoidosis diagnosed 2 years prior. Perihilar lymphadenopathy was evident on CXR and biopsy of a subcarinal lymph node demonstrated granulomatous inflammation. The sarcoidosis had not previously been treated. One year prior to presentation she had developed Mobitz type II heart block and a TTE and CMR at that time did not find evidence of CS. She also had a transient ischemic attack presenting as facial droop and myelopathy 3 weeks prior. A magnetic resonance imaging (MRI)/magnetic resonance angiogram of her brain did not find evidence of neurosarcoidosis or other abnormalities and a lumbar puncture showed cerebral spinal fluid protein of 0.76 with no white blood cells or oligoclonal bands. LV size and function was normal on TTE with no basal thinning of the intraventricular septum evident, and electrophysiological studies did not demonstrate inducible VT. Comorbidities included childhood epilepsy, type II diabetes mellitus, asthma, hypercholesterolemia, and tobacco smoking. Medications at the time of presentation were metformin, rosuvastatin, and aspirin.

Biochemical investigations including serum ACE of 24 (8 to 64), 1,25 vitamin D of 99 (60 to 100), and urine calcium of 6.6 nmol/L were all within normal limits. Dermatology and Ophthalmology review found no evidence of cutaneous or ocular sarcoidosis. A second CMR found normal biventricular size and systolic function and no late GAD enhancement seen in LV to suggest sarcoid involvement (Fig. 1bi). For the purposes of publication, the second CMR was subsequently reviewed by the same third party with specific expertise as used in Case 1. There was agreement that this CMR was within normal limits.

She proceeded to cardiac FDG-PET due to high clinical suspicion of CS despite two CMR scans within normal limits. Her BSL prior to scanning was 6.6 mmol/L following a 12-hour fast. The FDG-PET scan was suggestive of CS with active sites of focal myocardial inflammation in the basal anteroseptum, basal septum, and inferior walls (Fig. 1bii). She underwent successful permanent pacemaker (PPM)-AICD insertion due to risk of future VT and sudden cardiac death, and was commenced on metoprolol. She was commenced on prednisone 1 mg/kg orally administered daily and methotrexate. She was discharged and has had no further cardiac events. A repeat cardiac FDG-PET at 6 months demonstrated resolution of previously abnormal focal increased FDG accumulation in the LV consistent with resolution of areas of inflammation (see Fig. 1biii). The salient features of the two presented cases are shown in Table 3.

**Table 3:** Summary data for presented cases

Patient	Demographic	Duration of untreated pulmonary sarcoidosis	Presenting/additional cardiac complaint	Cardiac sarcoidosis treatment to date	Response to treatment
Patient 1	50 M	2 years	Dilated cardiomyopathy, CCF with LVEF 10–15%, non-sustained VT/AF	Prednisone Methotrexate	Improved LVEF to 53%. Complete resolution of cardiomegaly and FDG uptake on PET
Patient 2	37 F	2 years	CHB/Mobitz type II heart block	Prednisone Methotrexate PPM/AICD	Nil further cardiac events. Resolution of FDG uptake on PET

*AF* atrial fibrillation, *AICD* automated implantable cardiac defibrillator, *CCF* congestive cardiac failure, *CHB* complete heart block, *F* female, *FDG-PET* ^18^fluorodeoxyglucose-positron electron tomography, *M* male, *LVEF* left ventricular ejection fraction, *PPM* permanent pacemaker, *VT* ventricular tachycardia

## Discussion

FDG-PET is emerging as a useful tool for the diagnosis of CS and is thought to measure active macrophage infiltration and inflammation rather than fibrosis allowing for the detection of early disease. While CS classically demonstrates patchy myocardial uptake the pattern may also be diffuse or a combination of patchy on diffuse uptake [31]. Cardiac FDG-PET may have a slightly higher sensitivity for the diagnosis of CS (79 to 100%) as compared with CMR (76 to 100%), as the specificity has been reported as lower (39 to 100%) than CMR (78 to 92%) [21]. However, comparison of the diagnostic accuracy of FDG-PET versus CMR has been limited due to an imperfect reference standard and few studies that incorporate both modalities in large numbers of patients [32].

The limitations of FDG-PET for the diagnosis of CS include exposure to ionizing radiation, the need for careful preparation and fasting, and lower specificity. In addition, false negatives have been associated with scans taken where BSL was > 7.5mmol/L [3, 31, 33, 34].

Ohira *et al*. studied the diagnosis of CS in 21 patients who underwent both FDG-PET and CMR using the JMHW 2007 criteria as the gold standard [29, 35]. They found FDG-PET had a higher sensitivity for the diagnosis of CS (88% versus 75%) but lower specificity (39% versus 77%; [35]). More recent studies looking specifically at conduction delay have found patients with acute AV node block (such as Case 2) were more likely to have abnormal FDG-PET and normal CMR compared with patients with chronic mild CS presenting with other conduction abnormalities (33% compared with 0%) [36, 37]. It should be noted that image acquisition and interpretation of both CMR and FDG-PET for the diagnosis of CS can be challenging and requires specific expertise [10]. In terms of CMR, expertise is required in MRI set-up, quality control, scan supervision, and image interpretation. In fact, a re-review of CMR in the above cases found that the scan in Case 1 was indeterminate rather than a true negative due inadequate nulling of the myocardium in the delayed enhancement series. The resolution of FDG-PET avidity on serial imaging seen with the cases above is reassuring, especially given recent data demonstrating improved PET parameters correlate with improved LVEF and other clinical outcome measures [38, 39].

FDG-PET has been combined with ^82^rubidium perfusion scans to assess for adverse cardiac events in patients with confirmed or suspected CS [40]. In this study, patients with an abnormal FDG-PET and perfusion scan were at significantly higher risk of VT and sudden cardiac death (HR 3.9) independent of LVEF. There is an emerging role for combined FDG-PET and MRI modality for the diagnosis of CS; however, this has not been validated in large trials [21, 41].

There is a limited evidence base for the treatment of CS. Management is largely based on expert opinion and combines systemic immunosuppression with cardiac-specific therapies [3]. The aim of immunosuppression is to halt active granulomatous inflammation and to prevent fibrosis [32]. The optimal duration of therapy is unclear. Corticosteroids are the most commonly used first-line agent and have been shown to revert advanced AV block to first degree AV node block or sinus rhythm [42]. The evidence for corticosteroids improving ventricular arrhythmias is less clear [10].

Combination immune interventions that target different immune checkpoints are often used empirically. Methotrexate is the most commonly used second-line agent and this may additionally minimize harm from long-term steroid use. One study found no difference between corticosteroid treatment alone versus corticosteroids plus methotrexate in terms of LVEF or LV end diastolic diameter [43]. Azathioprine and mycophenolate have also been used successfully for the treatment of CS [18]. Tumor necrosis factor (TNF)-α blockade has been reported in case studies of patients with CS but should be used with caution in patients with LVEF < 35% because of an increased risk of adverse events as demonstrated in patients with CCF from other causes [44].

In addition to immunosuppression, cardiac-specific therapy may be indicated. PPMs are indicated for Mobitz type II and third degree AV block. General cardiac guidelines are inadequate in regard to indication for AICD insertion [45] and there is a Class I indication for AICD in patients with CS, ventricular arrhythmias, and LVEF ≤ 35% [10] (includes patient in Case 1 prior to treatment). The decision to insert an AICD must be weighed against potential risks including unnecessary shock in 10 to 30% of patients [3]. Anti-arrhythmic therapy and VT ablation may also be indicated. CCF should be managed as per protocols with diuretics, ACE inhibitor, and beta-blocker therapy. Appropriate patients with severe disease should be considered for cardiac transplant. Currently, sarcoidosis accounts for 1.5% of all cardiac transplants [46].

The prognosis of patients with CS is closely linked to LV function [42]. Immunosuppression improves prognosis and has been shown to increase LV function especially in those patients with moderately severe disease at diagnosis. The overall survival of patients with CS is 98% at 1 year and 84% at 10 years [47, 48]; however, this is markedly reduced in patients that have LVEF < 30% where 1-year and 10-year survival rates are 91% and 19%, respectively [48].

These two cases illustrate the importance of screening for asymptomatic CS and the need for vigilance in considering cardiac involvement in patients with extra-CS and cardiogenic symptoms. The cases highlight the heterogeneity of CS presentations and the difficulty in diagnosis due to risks of endomyocardial biopsy and lack of a singular accepted set of diagnostic criteria. Both patients presented with critical illness (severe CCF and CHB) and the diagnosis of CS was only made following FDG-PET as preceding CMR were unremarkable. Although neither patient underwent endomyocardial biopsy, both patients had confirmed histology for pulmonary sarcoidosis, and satisfied the HRS criteria, although interestingly the patient in Case 1 but not Case 2 qualifies on the JMHW guidelines due to the lack of inclusion of FDG-PET as an accepted criterion [29]. Both patients had presented 1 year prior with cardiac symptoms representing an opportunity for earlier diagnosis. Case 2 highlights the pitfalls in current HRS guidelines that state CMR *or* FDG-PET can be employed during diagnostic work-up. In fact, the diagnosis of CS in both cases would not have been made in the absence of FDG-PET, and important interventions including systemic immunosuppression may not have been implemented. These cases argue against a commonly held position that CMR is superior to FDG-PET for initial diagnosis due to high negative predictive value. We agree with the proposal by Hulten *et al*. that patients with negative CMR but persisting high clinical suspicion for CS should undergo subsequent FDG-PET [32].

The recovery of Case 1’s LVEF from < 15% to 53% following immunosuppression is remarkable and at odds with published data that suggest that LVEF does not substantially improve in patients with LVEF < 30% following immunosuppression [48]. Of interest, it has been previously thought that decreased LVEF < 30% is due to the high burden of LV scar tissue which is visible on CMR [3], however the patient in Case 1 would seem to argue against this due to the lack of delayed enhancement by traditional imaging. Furthermore Case 1 highlights the utility of serial FDG-PET during treatment and its correlation with LVEF [38].

Overall, these cases demonstrate that the diagnosis of CS facilitated by FDG-PET can lead to prompt management. Certainly, it appears likely that the immunosuppression together with cardiac-specific intervention has significantly decreased morbidity and improved prognosis in both cases.

## Conclusions

All patients diagnosed with sarcoidosis should undergo screening for cardiac involvement. At a minimum this should include cardiac history and examination, ECG, and TTE. If any of these demonstrate abnormalities then imaging studies are warranted. While CMR detects around 75% of cases, data suggest the sensitivity of FDG-PET may be 2 to 13% higher and may detect active myocardial inflammation in early disease prior to the formation of scar tissue. Patients who have normal CMR but where there is high suspicion for CS should undergo subsequent FDG-PET as diagnosis of this disease entity can allow for systemic immunosuppression and cardiac-specific therapy with a view to decreasing otherwise significant morbidity and mortality.

## Abbreviations

ACE: Angiotensin-converting enzyme
AF: Atrial fibrillation
AICD: Automated implantable cardiac defibrillator
ANA: Antinuclear antibodies
ANCA: Antineutrophil cytoplasmic antibodies
AV: Atrial-ventricular
BNP: Brain natriuretic peptide
BSL: Blood sugar level
CCF: Congestive cardiac failure
CCP: Cyclic citrullinated peptide
CHB: Complete heart block
CMR: Cardiac magnetic resonance imaging
CRP: C-reactive protein
CS: Cardiac sarcoidosis
CXR: Chest X-ray
DC: Direct current
dsDNA: Double-stranded DNA
EBUS: Endobronchial ultrasound
ECG: Electrocardiogram
ENA: Extractable nuclear antigens
FDG: ^18^F-fluorodeoxyglucose
FDG-PET: ^18^F-fluorodeoxyglucose-positron emission tomography
GAD: Gadolinium
HBV: Hepatitis B virus
HCV: Hepatitis C virus
HIV: Human immunodeficiency virus
HR: Hazard ratio
HRS: Heart Rhythm Society
IGRA: Interferon-gamma release assay
JMHW: Japanese Ministry of Health and Welfare
LV: Left ventricular
LVEF: Left ventricular ejection fraction
MRI: Magnetic resonance imaging
MTB: *Mycobacterium tuberculosis*
PCA: Posterior cerebral artery
PPM: Permanent pacemaker
RR: Reference range
SaO_2_: Arterial oxygen saturation
TNF: Tumor necrosis factor
TTE: Transthoracic echocardiogram
VT: Ventricular tachycardia
VZV: Varicella zoster virus
WASOG: World Association for Sarcoidosis and Other Granulomatous Disorders
